# Root lodging is a physical stress that changes gene expression from sucrose accumulation to degradation in sorghum

**DOI:** 10.1186/s12870-017-1218-9

**Published:** 2018-01-03

**Authors:** Hiroshi Mizuno, Shigemitsu Kasuga, Hiroyuki Kawahigashi

**Affiliations:** 1 Institute of Crop Science (NICS), National Agriculture and Food Research Organization, 2-1-2, Kannondai, Tsukuba, Ibaraki 305-8518 Japan; 2 Faculty of Agriculture, Shinshu University, 8304 Minami-minowa, Nagano, 399-4598 Japan

**Keywords:** Biofuel, RNA-seq, Stem, Sugar content, Sugar metabolism, Sugar transporter

## Abstract

**Background:**

Sorghum (*Sorghum bicolor* L.) is used as a raw material for biofuels because it accumulates sugars at high levels in the stem. Lodging of sorghum occurs when the soil is wet and very high winds blow across the field. In root lodging, the roots are pulled loose from the soil, causing the plant to fall over. Lodging reduces the yield of nonstructural carbohydrates. It is not yet clear which genes show changes in expression when sorghum falls over. We compared whole-gene expression in the mature stems of intact and lodged sorghum plants, with a focus on comparisons from the perspective of differences in sugar accumulation or degradation.

**Results:**

Lodging decreased sucrose content, starch content, and ratio of sucrose to total sugars in the stems of the sorghum cultivar SIL-05. Particular paralogs of *SWEET* and *TMT* family genes, which encode sucrose or hexose transporters, or both, were significantly highly expressed in intact or lodged sorghum stems. In intact stems, genes encoding the glucose-6-phosphate translocator, aquaporins, and enzymes involved in photosynthesis and starch synthesis were highly expressed. In lodged sorghum stems, expression of genes associated with sucrose or starch degradation or energy production was increased. Notably, expression of genes encoding enzymes catalyzing irreversible reactions and associated with the first steps of these metabolic pathways (e.g. *INV*, *SUS*, and hexokinase- and fructokinase-encoding genes) was significantly increased by lodging. Expression of *SUT*, *SPS*, and *SPP* was almost the same in intact and lodged sorghum.

**Conclusions:**

Specific paralogs of sucrose-associated genes involved in metabolic pathways and in membrane transport were expressed in the stems of sorghum SIL-05 at the full-ripe stage. Root lodging drastically changed the expression of these genes from sucrose accumulation to degradation. The changes in gene expression resulted in decreases in sugar content and in the proportion of sucrose to hexoses in the stems of lodged plants.

**Electronic supplementary material:**

The online version of this article (10.1186/s12870-017-1218-9) contains supplementary material, which is available to authorized users.

## Background

Biofuel productivity is affected by the type of biomass used and the efficiency of its conversion from plant-derived raw material to sugar [1]. The simplest and most cost-effective way to produce biofuels is to use materials containing large amounts of plant-derived sugars. Some plants, such as sorghum and sugarcane, uniquely accumulate sugar in their stems. The sorghum internode contains spacious apoplasts and vacuoles in which photosynthesized sugars are accumulated at high concentrations [2]. Sorghum therefore has high potential to become a feedstock for large-scale bioenergy production. However, its sugar content and composition vary depending on the cultivar [3–5]. Sorghums with a relatively high sugar content—so called sweet sorghums—have been bred for edible and industrial use [6].

In plants, an organ—such as the leaf—that supplies photosynthetic sucrose is called a source, and an organ—such as the seed and stem—that accepts them is called a sink. In a source organ, during sucrose biosynthesis, sucrose phosphate synthase (SPS) synthesizes sucrose-6-phosphate from UDP-glucose and fructose 6-phosphate; sucrose-6-phosphate is then dephosphorylated by sucrose phosphate phosphatase (SPP) to produce sucrose. In a sink organ, translocated sucrose is stored, or degraded and used for energy metabolism, organ formation, synthesis of stored carbohydrates such as monosaccharides and starch, or re-synthesis of sucrose. During sucrose metabolism, sucrose synthase (SUS) degrades sucrose to UDP-glucose and fructose, or invertase (INV) degrades sucrose to glucose and fructose. UDP-glucose is further converted to glucose-1-phosphate (G1P) by glucose-1-phosphate uridyltransferase; glucose is converted to glucose-6-phosphate (G6P) by hexokinase (HEX), and fructose is converted to fructose-6-phosphate by fructokinase (FRK). Nucleotide or phosphorylated sugars are activated forms of monosaccharides, which are precursors of various molecules such as starch. The starch biosynthesis pathway involves a step of ADP-α-D-glucose production by the action of glucose-1-phosphate adenylyltransferase and a repeating step of glycosyl transfer by starch synthase and further modification by the action of 1,4-α-glucan branching enzyme.

Plasma- or vacuolar-membrane-located sugar transporters also contribute to the distribution of sucrose. Sucrose is unloaded from the phloem; once it flows out into the apoplasts it is taken up by parenchymal cells and then accumulates in vacuoles [2, 7]. It is considered that sugars will eventually be exported transporter (SWEET) and sucrose transporter (SUT) transport sucrose, and tonoplast monosaccharide transporter (TMT) transports monosaccharides, but their substrate specificity has not been completely elucidated [8–14]. Aquaporins are needed to transport water into the cell or vacuole to counteract the effect of increasing osmotic pressure with increasing sugar content. Thus, high-level sugar accumulation is achieved by the combined function of various families of enzymes and transporters.

Sugars also act as signaling molecules that control metabolism and energy production in relation to the roles of sources and sinks. Such sugars include sucrose and fructose, and glucose and its derivatives G6P, G1P, and trehalose-6-phosphate (T6P) [15]. These signaling molecules regulate sugar utilization and starch metabolism and interact with other signaling pathways, including those mediated by plant hormones [15–17]. Direct sugar sensing and signaling occur via specific sugar-binding sensors. Candidates for sugar-binding sensors include SUT for sucrose; HEX and RGS (regulator of G-protein signaling) for glucose and its derivatives; fructose-1,6-bisphosphatase, FRK, and FLN (fructokinase-like protein) for fructose; and KIN10/11 (Arabidopsis protein kinase 10/11) and SnRK1 (sucrose non-fermenting related protein kinase 1) for T6P [15]. HEX, fructose-1,6-bisphosphatase, FRK, and SUT are dual-function molecules that act as both sugar sensors and enzymes involved in sugar metabolism or sugar transport [18].

In sorghum, comparisons of gene expression have been performed to identify systematically the critical genes that determine stem sugar content. For example, comparisons among diverse genotypes with different sugar contents (SSV74, SPV1616, R159, Atlas, Fremont, PI152611, AR2400, and PI455230) [19, 20] have revealed that changes in the expression levels of *SUT*, *SPS*, and *INV* are associated with variations in the sugar contents of different sorghum cultivars. Comparison of developmental stages has indicated that the expression levels of *INV* and *SUS* decrease after anthesis, when sucrose accumulation starts in the stem [21]. Although these comparisons have helped to elucidate the diversity of genes associated with sucrose metabolism, we still need to elucidate the critical genes regulating sugar accumulation and release.

However, a number of features of sorghum make it difficult to find genes associated with sugar content. Synthesis and degradation of sucrose occur at different rates in different types of cells simultaneously, and the organ structure of stems changes developmentally during long periods of sucrose accumulation [22, 23]. Sugar content is affected by environmental conditions [24]. Moreover, a number of phenotypic traits affect sugar content, namely plant height, total dry matter, grain yield, stem diameter, number of tillers per plant, juice weight, and flowering time [25, 26]. To identify the critical genes associated with sugar content, it is thus important, as far as is possible, to compare gene expression at the same developmental stage of the same tissue in the same cultivation environment. Therefore, we need to have an experimental method by which we can change and analyze gene expression from sucrose accumulation to degradation.

We hypothesized that root lodging (hereafter, lodging) is a physical stress that changes gene expression from sugar accumulation to degradation. Lodging of sorghum occurs when the soil is wet and very high winds blow across the field. The roots are pulled loose from the soil, causing the plant to fall over, although the stem remains intact. When lodging occurs in the early stages of the crop, the sorghum will usually re-stand, but the lodging reduces the yield of nonstructural carbohydrates [27]. It is not yet clear which genes show changes in expression when sorghum falls over. Here, we compared gene expression between intact and lodged sorghum of the cultivar SIL-05. SIL-05 accumulates sugars to relatively high levels in the stem [5] and is a promising candidate material for biofuel production. We used RNA-seq data to model the expression of specific genes supporting sugar accumulation and release in the stem. We discuss the characteristics of gene expression affected by lodging and the physiological factors in lodging stress that affect sugar metabolism.

## Results

### Lodging reduces Brix, sucrose percentage, and starch content

**Fig. 1:**
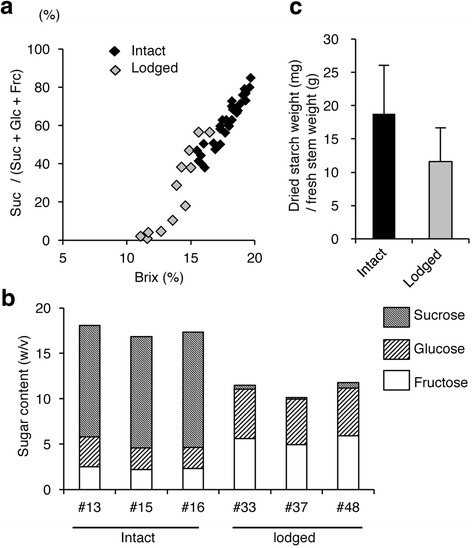
Differences in sugar content, sugar composition, and starch content of stem between lodged and intact plants. **a** Brix and sucrose percentage. Horizontal axis represents Brix and vertical axis represents sucrose weight as a percentage of the total weights of sucrose, glucose, and fructose combined. Data on 36 intact plants (black) and 12 lodged plants (gray) are plotted. **b** Sugar composition. The contents (weight/volume) of sucrose, glucose, and fructose of three individual plants of intact (# 13, # 15, # 16) and lodged (# 33, # 37, # 48) sorghum are shown. **c** Starch content. The mean and standard deviation of the values of the starch content (dried starch weight (mg)/stem fresh weight (g)) for three individual intact (# 13, # 15, # 16) and lodged (# 33, # 37, # 48) plants are shown

We grew 48 plants of the sorghum cultivar SIL-05 in a field. Twelve of the plants lodged at the ear-heading stage and were still lodged at the time of full seed ripening. Thirty-six plants remained upright. The Brix of the stem ranged from 11.1% up to 19.7% across all plants, but the lodged plants had relatively low Brix values (Fig. 1a). The sucrose ratio (the sucrose content as a percentage of the sum of sucrose, glucose, and fructose weights) ranged from 0% up to 84.9% (Fig. 1a). The sucrose content of the intact plants was greater than that of the lodged plants, whereas the glucose and fructose contents were lower (Fig. 1a). We then selected three independent typical intact (#13, #15, #16) and lodged (# 33, # 37, # 48) plants for further analysis (Fig. 1b). The average Brix of the intact plants was 18.7%, whereas that of the lodged plants was 11.8% (Fig. 1b), indicating that sucrose had accumulated to high levels in the stems of the intact plants. The sucrose ratio in the intact plants was 71.3%, whereas that in the lodged plants was 3.7% (Fig. 1b). Both the glucose content and the fructose content in the lodged plants were almost double those in the intact plants; thus, the Brix values of the lodged plants were explained nearly entirely by the plants’ hexose contents. Starch content tended to be higher in intact plants than in lodged ones (Fig. 1c). There was slight but significant difference in raw panicle weight (intact > lodged), but there were no significant differences in plant height, stem diameter, or raw stem weight between lodged and intact plants (Additional file 1: Figure S1).

### Identification of differentially expressed genes by RNA-seq

**Fig. 2:**
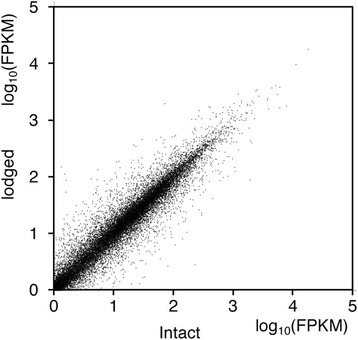
Changes in gene expression level with lodging. Fragments per kilobase of exon per million mapped sequence reads (FPKM) of 27,608 genes were plotted for lodged plants (vertical axis) and intact plants (horizontal axis). Values are log_10_ of average FPKM calculated from three individual plants: intact (# 13, # 15, # 16) or lodged (# 33, # 37, # 48)

RNA extracted from intact and lodged plants was sequenced by using a next-generation sequencer. Fragments per kilobase of exon per million mapped sequence reads (FPKM) for each gene were compared between intact and lodged plants (Fig. 2). Of a total of 27,608 genes, 1771 were significantly highly expressed in intact plants and 1866 were significantly highly expressed in lodged plants (Additional file 2: Table S1).

### Overview of metabolic pathways activated in intact and lodged sorghum plants

**Fig. 3:**
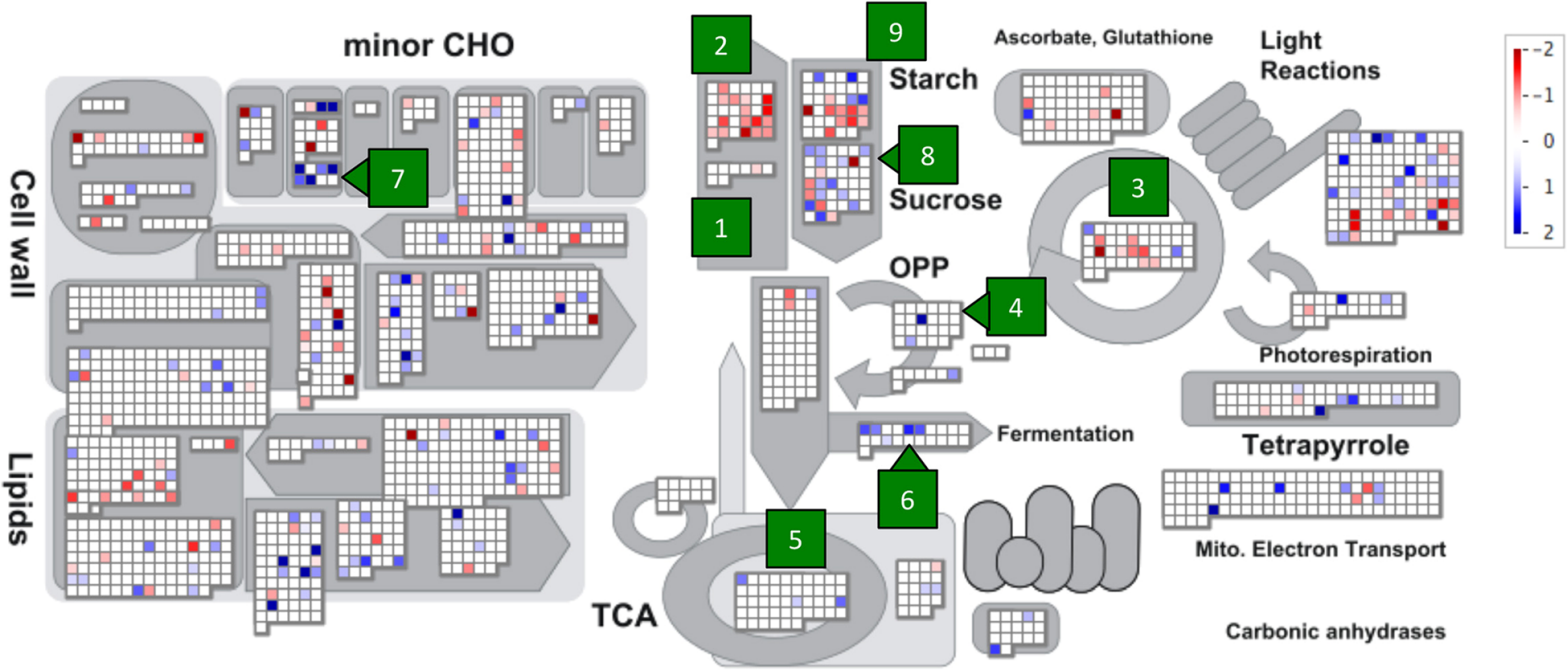
Relative expression levels of metabolism-associated genes in lodged and intact plants. Each box represents 2778 predicted genes involved in metabolic pathways; these genes were annotated with MapMan software. Colors represent relative expression levels: significantly higher in intact plants (red); significantly higher in lodged plants (blue); no significant difference (white). Green numbers represent metabolic pathways discussed in the main text. 1: sucrose synthesis; 2: starch synthesis; 3: Calvin cycle; 4: pentose phosphate pathway; 5: TCA cycle; 6: fermentation; 7: trehalose synthesis; 8: sucrose degradation; 9: starch degradation

The relative FPKM of lodged and intact plants were mapped on the metabolic pathways. In intact plants, expression of genes involved in sucrose or starch synthesis or the Calvin cycle was high (Fig. 3: pathways 1 to 3). In lodged plants, expression of genes involved in the pentose phosphate pathway, the TCA cycle, the fermentation pathway, and the trehalose pathway (Fig. 3: pathways 4 to 7) was high. Different paralogs involved in sucrose degradation or starch degradation were expressed at high levels in intact or lodged plants (Fig. 3, pathways 8 and 9).

### Expression of genes encoding enzymes involved in sucrose and starch metabolic pathways

#### Sucrose synthesis

**Fig. 4:**
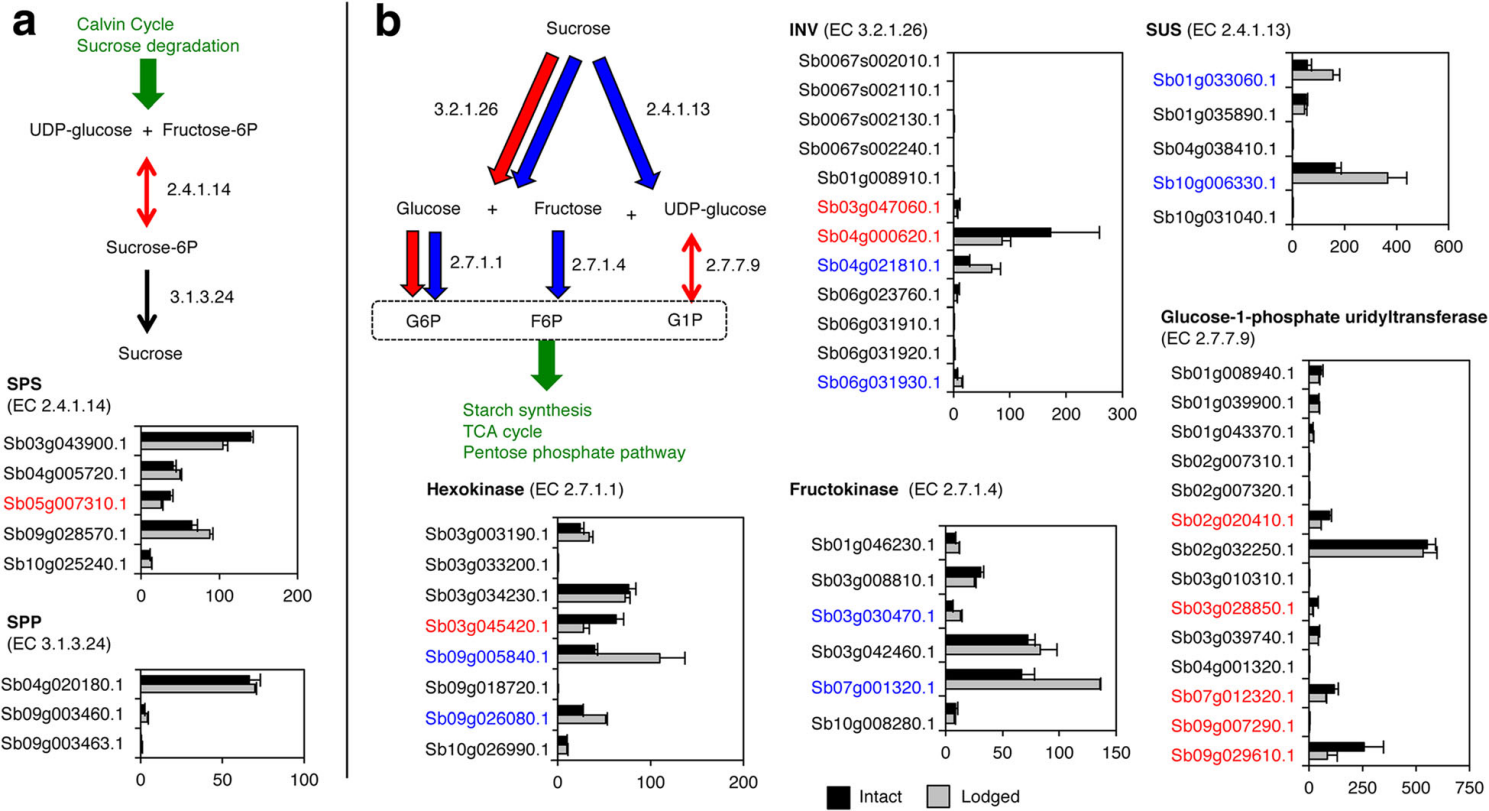
Expression levels of genes involved in sucrose metabolism. **a** Sucrose synthesis. **b** Sucrose degradation. One arrowhead indicates an irreversible reaction, and double arrowheads indicate a reversible reaction. Colors of arrows and of gene names represent relative gene expression levels: significantly higher in intact plants (red); significantly higher in lodged plants (blue); no significant difference (black). Graphs indicate average numbers of FPKM (fragments per kilobase of exon per million mapped sequence reads) of intact (black) or lodged (gray) plants and standard errors from three individual plants. Green arrow represents the link from other metabolic pathways

Sucrose biosynthesis is a two-step enzymatic reaction starting from UDP-glucose. Expression of the genes involved in sucrose biosynthesis was barely changed by lodging. Of five *SPS* and three *SPP* paralogs, only the expression of one *SPS* (Sb05g007310.1) was significantly higher in intact than in lodged sorghum stem (Fig. 4a).

#### Sucrose degradation

Sucrose degradation is catalyzed by either INV (EC 3.2.1.26) or SUS (EC 2.4.1.13). In lodged plants, expression of *INV* (Sb04g021810.1 and Sb06g031930.1) and *SUS* (Sb01g033060.1 and Sb10g006330.1) was significantly higher than in intact plants (Fig. 4b). Expression of the genes encoding hexokinase (Sb09g005840.1 and Sb09g026080.1) and fructokinase (Sb03g030470.1 and Sb07g001320.1) was also significantly higher in lodged stems (Fig. 4b).

#### Starch synthesis

**Fig. 5:**
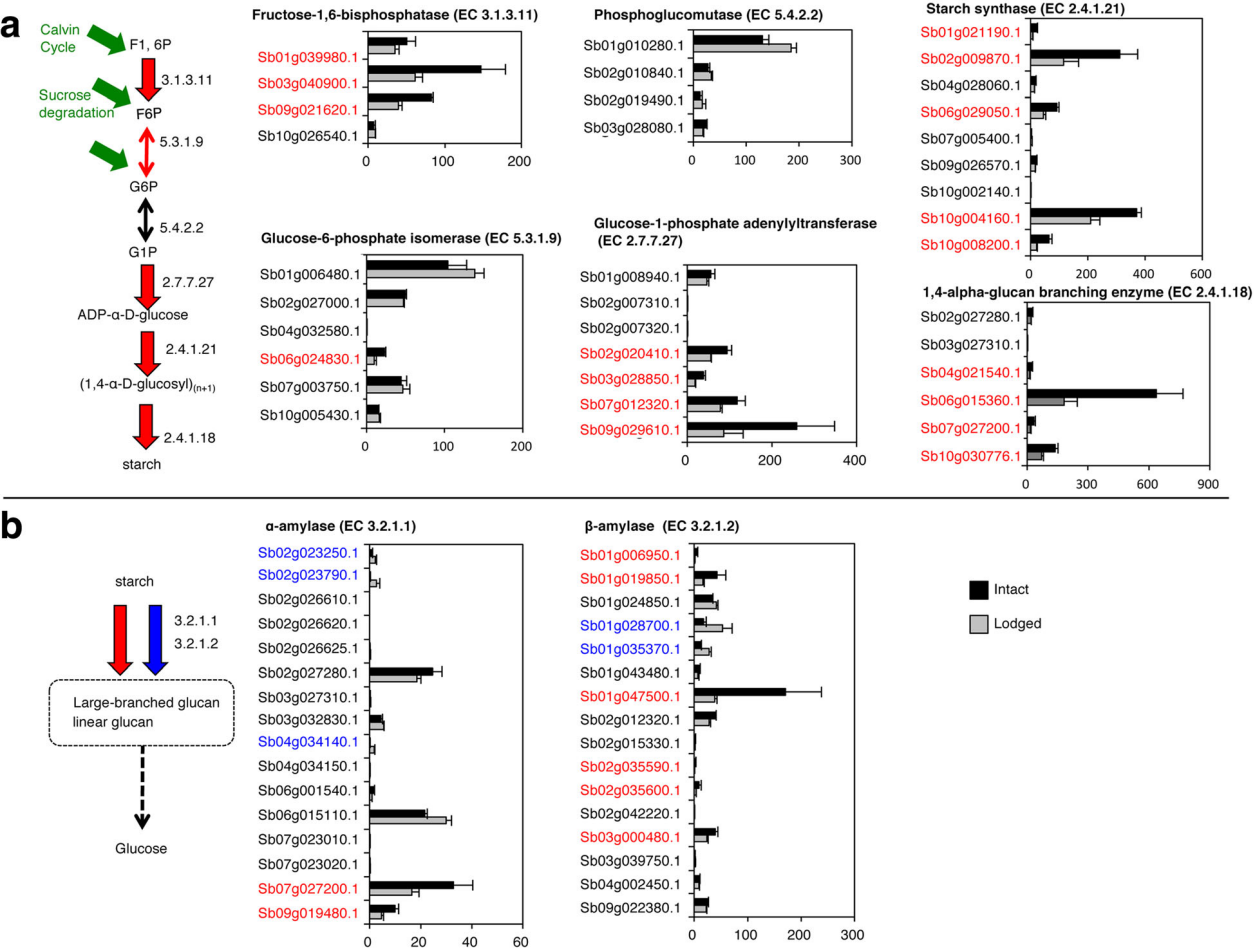
Expression levels of genes involved in starch metabolism. **a** Starch synthesis. **b** Starch degradation. Designations are as in Fig. 4

Genes encoding enzymes involved in starch biosynthesis steps were expressed at significantly higher levels in the stems of intact sorghum than in those of lodged plants (Fig. 5a). Notably, genes encoding fructose-1,6-biphosphatase (EC 3.1.3.11), glucose-1-phosphate adenylyltransferase (EC 2.7.7.27), starch synthase (EC 2.4.1.21) and 1,4-alpha glucan branching enzyme (EC 2.4.1.18) were expressed at higher levels in intact plants than in lodged plants.

#### Starch degradation

There are 16 putative genes encoding α-amylases and 16 β-amylases in the sorghum genome. Different paralogs putatively encoding α-amylases or β-amylases were expressed at high levels in intact or lodged sorghum (Fig. 5b).

#### Photosynthesis

In lodged plants, expression of genes involved in the synthesis of sucrose via photosynthesis was decreased. In the case of the Calvin cycle, expression of genes for phosphoribulokinase (EC 2.7.1.19; Sb04g030950.1), fructose-1,6-bisphosphatase (EC 3.1.3.11; Sb03g040900.1), and fructose-bisphosphate aldolase (EC 4.1.2.13; Sb05g004590.1) was highly (FPKM > 100) and significantly decreased by lodging (Additional file 3: Figure S2). Expression of genes associated with C4 photosynthesis (encoding phosphoenolpyruvate carboxylase (EC 4.1.1.31), malate dehydrogenase (EC 1.1.1.82), and pyruvate-phosphate dikinase (EC 2.7.9.1)) was affected little by lodging (Additional file 4: Figure S3).

### Construction of models of expression of critical genes associated with sugar storage and release in stems (intact or lodged)

#### Stem (intact)

**Fig. 6:**
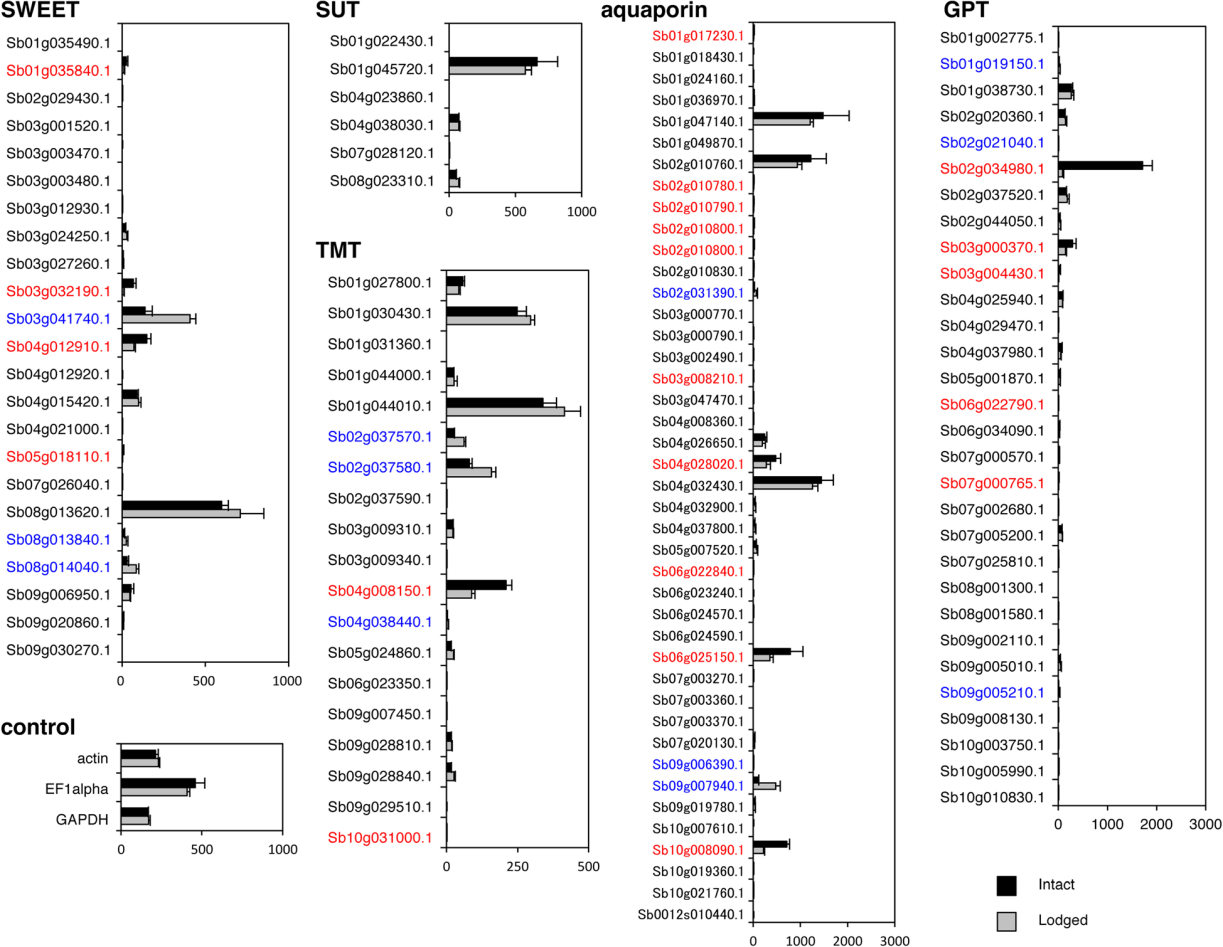
Expression of putative transporter genes involved in sugar accumulation or release: 23 *SWEET*, six *SUT*, 19 *TMT*, 30 *GPT*, and 42 aquaporin genes. Constitutively expressed control genes, *actin* (Sb08g003970), *elongation factor 1-alpha* (*EF1alpha*; Sb10g023330), and *glyceraldehyde 3-phosphate dehydrogenase* (*GAPDH*; Sb10g029870), are also shown. Designations are as in Fig. 4

Sucrose is translocated from the leaves into the sink organ, namely the stem. Sucrose is accumulated in the vacuoles or is degraded to hexoses in the cytosol. These hexoses are stored or used for starch synthesis. Among the genes for sucrose transporters, the expression of *SWEET* genes (Sb01g035840.1, Sb03g032190.1, Sb04g012910.1, Sb05g018110.1) was significantly higher in intact stems than in lodged ones (Fig. 6). Both *SWEET4–3* (Sb04g015420.1.1) and *SbSUT1* (Sb01g045720.1.1) were highly expressed in the stem at the sucrose-accumulation stage [28], but their expression was not changed by lodging (Fig. 6). Among the genes for hexose transporters, *TMT*s (Sb01g030430.1, Sb01g044010.1, Sb04g008150.1) were expressed highly in the stem; Sb04g008150.1 was expressed at significantly higher levels in intact plants than in lodged ones (Fig. 6).

For water transport, 10 putative aquaporin genes were expressed at significantly higher levels in intact than in lodged plants, and three of them (Sb04g028020.1, Sb06g025150.1 and Sb10g008090.1) were highly expressed (Fig. 6). Three aquaporin genes (Sb01g047140.1, Sb02g010760.1, and Sb04g032430.1) were highly expressed at the same levels in both intact and lodged stems (Fig. 6).

**Fig. 7:**
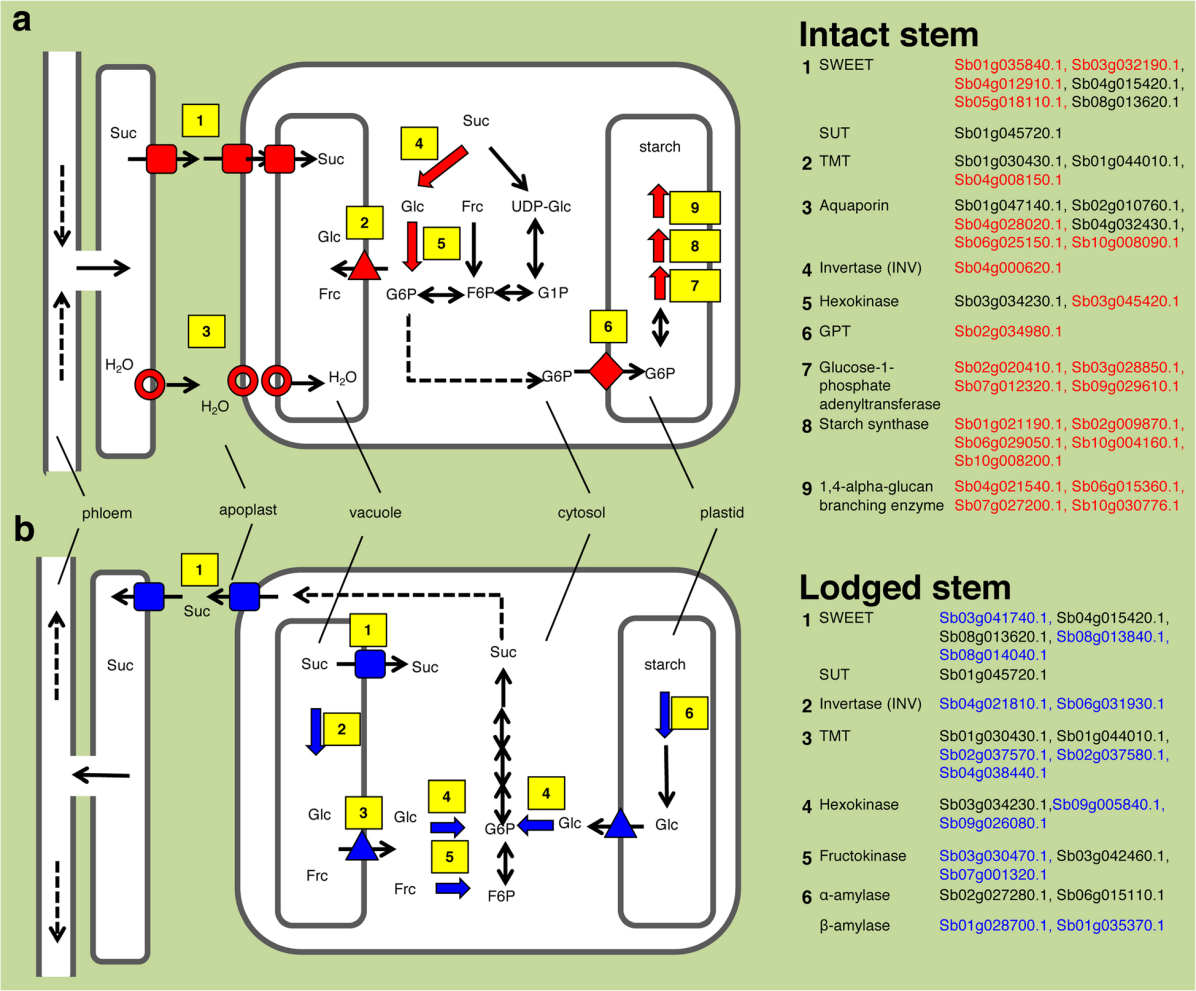
Representative genes involved in sugar accumulation or release. **a** Model of intact sorghum. Sucrose (Suc) is accumulated in the vacuoles or is degraded to glucose (Glc) and fructose (Frc) in the cytosol. These hexoses are stored or used for starch synthesis. Genes for sucrose accumulation were highly expressed in intact stems. **b** Model of lodged sorghum. Sucrose stored in vacuoles is translocated to other tissues. Sucrose is synthesized from stored starch or hexoses. Genes for release of stored sucrose to other tissues were expressed in the lodged stem. Illustrations on the left show schematically the enzymatic reactions or transport steps involved in sugar accumulation or release. Yellow numbers correspond to the numbers in the tables at right. In these tables, genes within each family with expression levels that changed with lodging (red: significantly higher in intact plants; blue: significantly higher in lodged plants) and genes that were expressed at high levels but not differentially between intact and lodged plants (black) are listed

Expression of *SPS* and *SPP* genes was barely changed by lodging (Fig. 4). In the case of starch synthesis, 4 of 7 genes encoding glucose-1-phosphate adenylyltransferases, 5 of 9 genes encoding starch synthases, and 4 of 6 genes encoding 1,4-α-glucan branching enzymes were expressed at significantly higher levels in the intact stem than in the lodged stem (Fig. 5a). One *glucose 6-phosphate/phosphate translocator2* (*GPT2*) (Sb02g034980.1) was expressed at much higher levels in intact than in lodged stems (Fig. 6). These findings were summarized as a model in Fig. 7a.

#### Stem (lodged)

Sucrose stored or synthesized is translocated to other tissues. For sucrose transport, the expression of *SWEET*s (Sb03g041740.1, Sb08g013840.1, Sb08g014040.1) was increased by lodging; the expression level of *SbSWEET3–8* (Sb03g041740.1) was especially high (Fig. 6). For degradation of stored sucrose, expression of *INV* (Sb04g021810.1, Sb06g031930.1) was significantly higher in lodged stems than in intact ones (Fig. 4b). For hexose transport, expression of *TMT*s (Sb02g037570.1, Sb02g037580.1, Sb04g038440.1) was significantly increased by lodging (Fig. 6). For stored starch degradation, expression of some putative genes encoding α-amylases and β-amylases was high (Fig. 5b). For hexose phosphorylation, expression of some genes encoding hexokinases and fructokinases was increased significantly in lodged plants (Fig. 4b). We consider that these phosphorylated sugars eventually become sucrose and are translocated to other organs for use in energy-releasing glycolysis. The levels of expression of these genes were consistent with our biochemical quantification that lodged stems had lower sugar and starch contents and lower sucrose percentages than intact stems (Fig. 1a, b). These findings were summarized as a model in Fig. 7b.

## Discussion

### Characteristics of sugar-associated genes affected by lodging

To our knowledge, our study is the first to compare gene expression in the stems of intact sorghum plants with that in the stems of lodged sorghum plants. We focused on comparisons from the perspective of differences in sugar accumulation or degradation, instead of comparisons of different organs, developmental stages, or genetic variations with different sugar contents [19, 20, 29, 30]. Lodging was a physical stress greatly affecting the expression of sugar-associated genes at the same developmental stage, in the same tissue, in field environments that were as similar as possible. We revealed that lodging changed sugar content and composition in the stems of the sorghum SIL-05 (Fig. 1). This was consistent with the results of our transcriptome analysis indicating that gene expression was changed from sucrose synthesis to degradation by lodging; in other words, cell status changed from energy storage to consumption by lodging (Figs. 2, 3, 4, 5, 6 and 7). We thus concluded that lodging was a physical stress that changes gene expression from sugar accumulation to degradation.

Changes in stem-specific gene expression reflect the transport of molecules to sugar storage compartments such as the apoplast and vacuoles in stem. SWEET or TMT paralogs differentially expressed upon lodging (Fig. 6) might transport sugars to and from stem-specific compartments (uptake or efflux) in accordance with changes in the demand for, and supply of, cell energy (Fig. 7). In contrast, the expression levels of *SUT*, *SPS*, and *SPP* were almost the same in intact and lodged plants (Figs. 4a and 6). This suggests that expression of *SUT*, *SPS*, and *SPP* is necessary for sucrose accumulation but does not change during the regulation of sugar metabolism. The expression level of *SUT* among different cultivars with different sugar content is controversial: it differs between Rio and BTx623 [31], but it does not differ between Wray and Macia [32]. We therefore consider that expression of the differentially expressed *SWEET* or *TMT* genes is regulated respectively to accumulate or release sugar in the stem of SIL-05.

Whether stored molecules are kept or degraded is often determined by the action of the first enzymes in the metabolic pathway, as usually this step is irreversible. INV enzymes are the first to degrade sucrose. At the early stage of sucrose accumulation (1 to 36 days after heading), *INV*s were barely expressed in the stem [28]. *INV* expression was upregulated by lodging (Fig. 4b). These data suggest that INV activity does not occur in the stem at the sucrose accumulation stage, but that INV activity is required to generate energy in the case of lodging. This is strongly supported by the finding that *INV* expression rapidly decreases after anthesis in the sorghum Della [21].

To convert stored hexoses into various molecules, they must first be converted to activated intermediates. Both hexokinase and fructokinase phosphorylate hexoses to produce high-energy intermediates, and those genes were highly expressed in lodged sorghum (Fig. 4b). We therefore consider that lodging-induced expression of these genes encoding hexokinases and fructokinases helps supply stored sugars to sugar metabolic pathways such as glycolysis.

As many enzymes involved in sugar synthesis and degradation are the same, the direction of the pathway is determined by a limited number of irreversible enzymatic reactions. In the pathway from hexoses to pyruvate for the TCA cycle and its reverse pathway, there are two irreversible steps. A total of four enzymes are responsible for these irreversible reactions, namely 6-phosphofructokinase (EC 2.7.1.11), fructose-1,6-bisphosphatase (EC 3.1.3.11), phosphoenolpyruvate synthase (EC 2.7.9.2), and pyruvate kinase (EC 2.7.1.40). Of the genes encoding these four, only the genes encoding fructose-1,6-bisphosphatase were expressed at significantly higher levels in intact than in lodged sorghum stems (Fig. 5a; Additional file 5: Figure S4). We therefore consider that the level of production of fructose-1,6-bisphosphatase helps to determine the direction of sucrose synthesis or glycolysis in photosynthetic cells.

Our molecular-based knowledge of sink organs has been obtained mainly by using the seeds or tubers of model plants such as Arabidopsis, rice, maize, and potato [1, 10, 12–14]. However, as these model plants do not accumulate sugar at high levels in the stem, we consider that the sugar-associated genes of these model plants do not functionally correspond to orthologs in sorghum that are critical for stem sugar accumulation. The sugar-transport mechanism of the sugarcane internode is symplastic unloading [33], which differs from the apoplastic unloading in sorghum [2]. In addition, the sugarcane genome is very complex owing to its high polyploidy and aneuploidy, with 10 to 12 copies of homeologous genes [34]; this is another reason why sugarcane genes do not necessarily correspond to sorghum orthologs critical for stem sugar accumulation. Sucrose-metabolism-associated gene families (*SUS*, *SPS*, *SPP*, and *glucose-1-phosphate uridylyltransferase*) have diversified because of differences in evolutionary history, as their host genomes have experienced differential rates of whole-genome duplication, tandem and segmental duplication, or mobile-element-mediated gene gain and loss [35]. Transporter genes are more diverse than sugar metabolism genes, and the evolutionary divergence of sugar transporter genes is associated with differences in the degree of sugar accumulation in the storage tissues of grasses and eudicots [36]. Moreover, *SbSWEET4–3*, which is constitutively highly expressed in the stem, was generated after the divergence of rice and sorghum [28]. Thus, the sugar-associated genes found in this study might have evolved as specific to sorghum.

### Physiological factors in lodging stress that affect sugar metabolism

Access to light may be hindered in lodged plants. In the lodged plants, expression of genes associated with the Calvin cycle decreased (Additional file 3: Figure S2). This supports a decline in photosynthetic capacity of the lodged plants. We consider that, unlike the intact plants, the lodged plants were in a state of energy shortage; accumulated sugars and starch were therefore degraded to compensate for this shortage.

Because, in the lodged plants, oxygen consumed through respiration was not adequately supplied, the intracellular oxygen concentration of the tissues of the lodged plants may have decreased locally, resulting in hypoxia. The expression of genes putatively involved in fermentation (*alcohol dehydrogenase* (*ADH*), *pyruvate decarboxylase* and *L-lactate dehydrogenase*) was increased by lodging (Fig. 3). The expression of *ADH* genes in maize increases in hypoxia [37]. The putative sorghum orthologs (Sb01g008730, Sb05g009350) of the maize hypoxia-induced *ADH1*/*2* were relatively highly expressed in lodged sorghum (Additional file 2: Table S1). We therefore consider that fermentation was activated under intracellular anaerobic conditions in lodged plants.

A change caused in the concentration of sugar molecules by lodging may act as a signal to regulate sugar metabolism and energy production in the whole plant. In the lodged plants, the proportion of hexoses (glucose, fructose) to sucrose was increased (Fig. 1). The changes in expression levels of *INV*, *SUS*, and the genes encoding hexokinase, fructokinase, and glucose-1-phosphate uridyltransferase with lodging (Fig. 4b) may thus contribute to the regulation of sugar metabolism and energy production in sources and sinks through the generation and/or the sensing of signaling molecules (glucose, fructose, G6P, and G1P). T6P may be another signaling molecule [16, 17]. We found that genes involved in trehalose metabolism were upregulated by lodging (Fig. 3): the expression of 6 of 15 putative T6P synthase (TPS)-encoding genes (Sb09g025660.1, Sb07g020270.1, Sb07g021920.1, Sb04g035560.1, Sb02g023610.1, Sb02g024140.1) was significantly increased by lodging (Additional file 2: Table S1). Although we did not examine the occurrence of T6P, the increase in *TPS* expression suggests that the concentration of T6P increased in lodged plants. Identification of sugar molecules that function as signals in sorghum and of the corresponding binding sensors is an important future goal.

Other studies have demonstrated how physical stress can change sugar metabolism. Wounding induces the expression of *INV*, followed by hexose accumulation and post-harvest sucrose loss, in sugar beet [38]. Drought or salt stress can induce the expression of sugar transporter genes in Arabidopsis [39]. Comparison with the responses to other types of physical stress will elucidate the diversity of the physical stresses that affect the expression of genes involved in sugar metabolism.

## Conclusions

Specific paralogs of sucrose-associated genes involved in metabolic pathways and in membrane transport were expressed in the stems of sorghum SIL-05 at the full-ripe stage. Root lodging drastically changed the expression of these genes from sucrose accumulation to degradation. The changes in gene expression resulted in decreases in sugar content and in the proportion of sucrose to hexoses in the stems of lodged plants.

## Methods

### Plant materials and quantification of sugar and starch contents

Seeds of sorghum cultivar SIL-05 were sown on 26 May 2012 in a field at Shinshu University in Nagano, Japan. Weather-induced lodging occurred in 12 of the 48 plants just after ear-heading in late August 2012. The lodged plants were still lodged at the time of sampling of their internodes on 10 October 2012. Internodes located in the central part of each plant were collected. The collected internodes were completely developed and the seeds were fully ripe. For quantification of sugars, juice at the internode was obtained by using a French press. The Brix of the sorghum juice was measured with a PAL-1 refractometer (Atago, Tokyo, Japan). Sugar contents (sucrose, glucose, and fructose) were measured by capillary electrophoresis [5]. For quantification of starch, frozen internodes were powdered, shaken with 0.2% NaOH for 30 min, and stored overnight at 4 °C. The extract was filtered through gauze to remove the insoluble residual fibers. The filtered extract was centrifuged and the starch pellet was washed stepwise with 0.2% NaOH, distilled water, and methanol and then dried. The dried starch weight (mg)/stem fresh weight (g) ratios were compared between intact and lodged plants.

### RNA-seq

Sorghum stems were immediately frozen in liquid nitrogen, fragmented, and mixed to minimize the effect of transcriptome unevenness among stem regions. RNA was extracted with an RNeasy plant kit (Qiagen, Hilden, Germany). RNA quality was calculated by using the Bioanalyzer 2100 algorithm (Agilent Technologies, Palo Alto, CA, USA); high-quality (RNA integrity number > 8) RNA was used for RNA-seq. cDNAs were synthesized from RNAs [40, 41] and 100-bp reads were obtained by using an Illumina Hiseq2000 sequencer (Illumina, San Diego, CA, USA). Low-quality nucleotides (<Q15) from both the 5′- and the 3′-ends were trimmed by using btrim [42]. Reads were aligned to the reference genome of BTx623 (Sbicolor_v1.4_79) [43] by using TopHat version 2.0.4 [44]. Differentially expressed genes were identified by using Cuffdiff version 2.2.0 [45], with the gene models annotated in Sbicolor ver1.4 [46].

### Mapping genes on metabolic pathways

MapMan (3.5.1 R2) software [47] was used to map the ratio of expression (lodged to intact) of each gene to known metabolic pathways. The log_2_ value was calculated after adding 1 to the FPKM of each gene. Lists of enzyme-encoding genes belonging to each metabolic pathway were downloaded from SorghumCyc ver 1.1 [48] and manually modified.

## Additional files

Additional file 1: Figure S1. Comparison of sizes and weights in intact and lodged sorghum. Average plant height, stem diameter, raw stem weight, and raw panicle weight of 36 intact plants and 12 lodged plants are shown. (PDF 27 kb)

Additional file 2: Table S1. Comparison of expression levels of sorghum genes in intact and lodged stems. Columns show the following, from the left: gene name (gene_id), chromosomal position (locus), average FPKM (fragments per kilobase of exon per million mapped sequence reads) of three intact sorghum plants (fpkm_intact), average FPKM of three lodged sorghum plants (fpkm_lodged), log_2_ value of fold change from lodged sorghum to intact sorghum (log2_ratio), *P*-value (p_value), q-value (q_value), and statistical significance (yes/no). (XLSX 2174 kb)

Additional file 3: Figure S2. Comparison of expression levels of genes involved in the Calvin cycle in intact and lodged stems. Columns show the following, from the left: gene name, reaction EC, average FPKM of three intact plants (FPKM_intact), average FPKM of three lodged sorghum plants (FPKM_lodged), and statistical significance (yes/no). (PDF 42 kb)

Additional file 4: Figure S3. Comparison of expression levels of genes involved in C4 photosynthesis in intact and lodged stems. Designations are as in Additional file 3: Figure S2. (PDF 37 kb)

Additional file 5: Figure S4. Expression of genes involved in irreversible steps of sugar metabolism. One arrowhead indicates an irreversible reaction, and double arrowheads indicate a reversible reaction. Colors of arrows and of gene names represent relative gene expression levels: significantly higher in intact plants (red); no significant difference (black). Graphs indicate average FPKM of intact (black) or lodged (gray) plants and standard errors from three individual plants. (PDF 481 kb)

## Abbreviations

FPKM: Fragments per kilobase of exon per million mapped sequence reads
FRK: Fructokinase
G1P: Glucose-1-phosphate
G6P: Glucose-6-phosphate
GPT: Glucose 6-phosphate/phosphate translocator
HEX: Hexokinase
INV: Invertase
SPP: Sucrose phosphate phosphatase
SPS: Sucrose phosphate synthase
SUS: Sucrose synthase
SUT: Sucrose transporter
SWEET: Sugars will eventually be exported transporters
T6P: Trehalose-6-phosphate
TMT: Tonoplast monosaccharide transporter
TPS: T6P synthase
