# Supplementary figures and images for: Root lodging is a physical stress that changes gene expression from sucrose accumulation to degradation in sorghum
Source: BMC Plant Biol. 2018 Jan 3;18:2. doi: 10.1186/s12870-017-1218-9 (PMC5751775; doi:10.1186/s12870-017-1218-9)

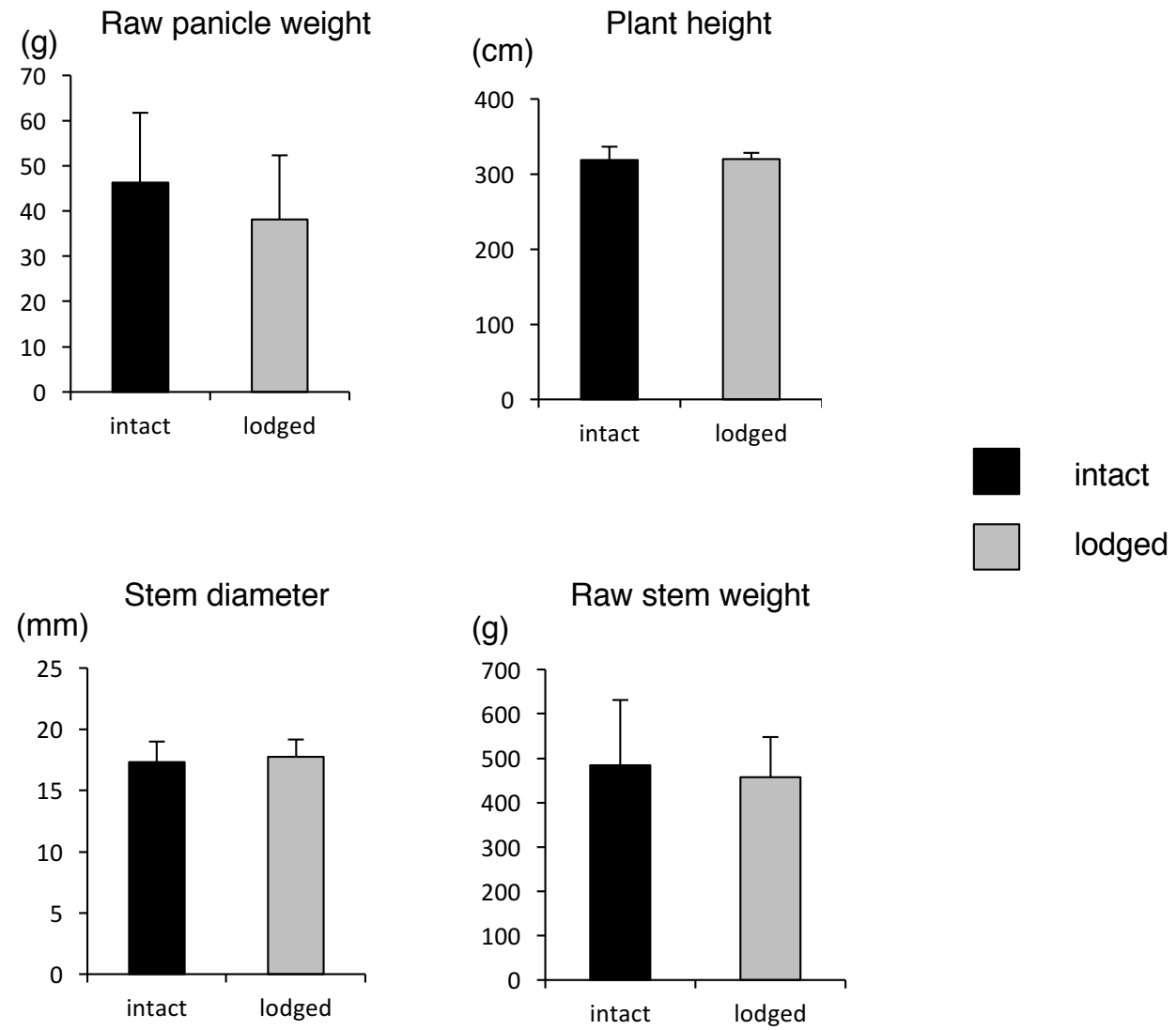

Figure S1

Supplement: Supplementary file 1 — Comparison of sizes and weights in intact and lodged sorghum. Average plant height, stem diameter, raw stem weight, and raw panicle weight of 36 intact plants and 12 lodged plants are shown. (PDF 27 kb) [file 12870_2017_1218_MOESM1_ESM.pdf]

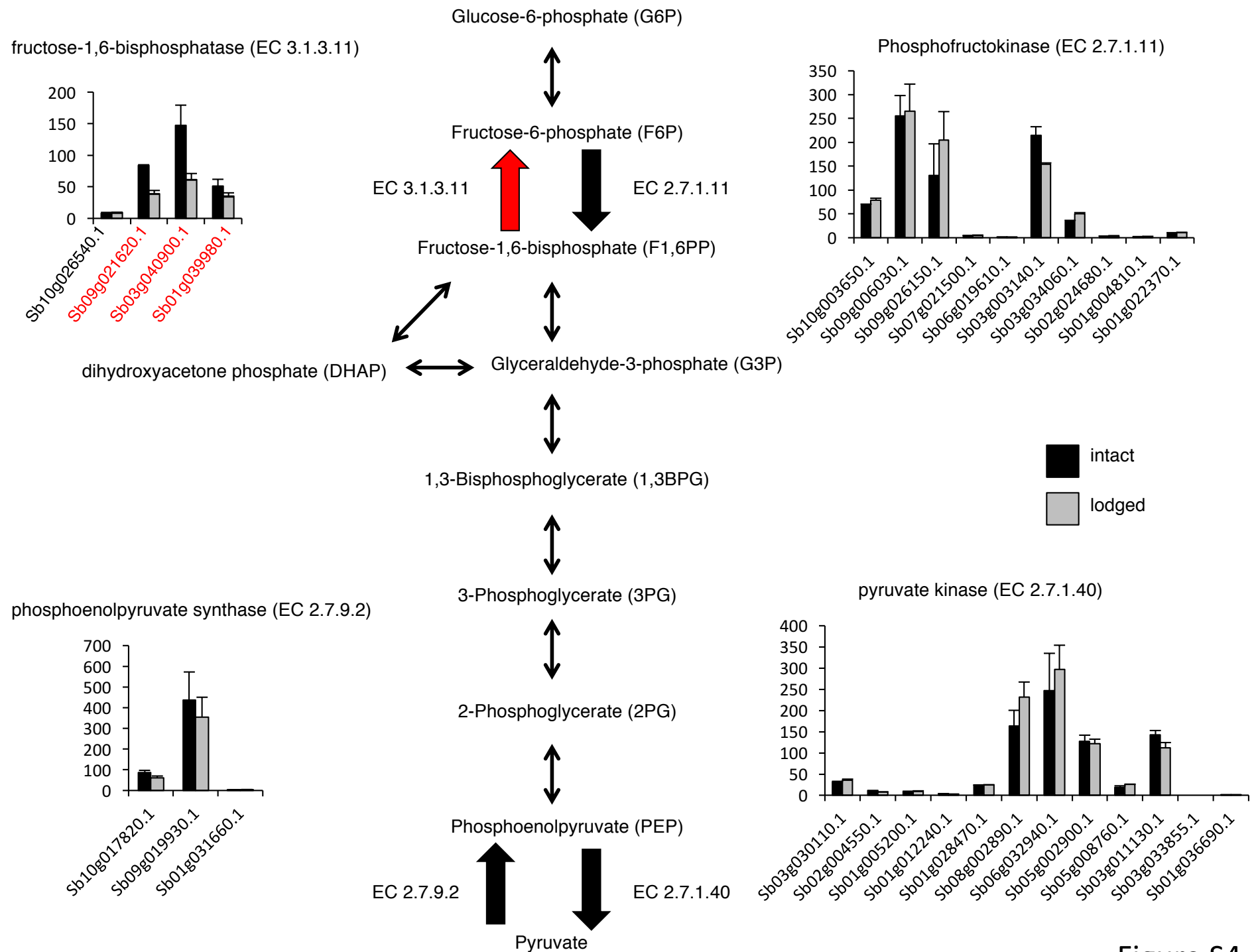

Figure S4

Supplement: Supplementary file 5 — Expression of genes involved in irreversible steps of sugar metabolism. One arrowhead indicates an irreversible reaction, and double arrowheads indicate a reversible reaction. Colors of arrows and of gene names represent relative gene expression levels: significantly higher in intact plants (red); no significant difference (black). Graphs indicate average FPKM of intact (black) or lodged (gray) plants and standard errors from three individual plants. (PDF 481 kb) [file 12870_2017_1218_MOESM5_ESM.pdf]
