# Supplementary material for: Root lodging is a physical stress that changes gene expression from sucrose accumulation to degradation in sorghum
Source: BMC Plant Biol. 2018 Jan 3;18:2. doi: 10.1186/s12870-017-1218-9 (PMC5751775; doi:10.1186/s12870-017-1218-9)
Supplement: Supplementary file 3 — Comparison of expression levels of genes involved in the Calvin cycle in intact and lodged stems. Columns show the following, from the left: gene name, reaction EC, average FPKM of three intact plants (FPKM_intact), average FPKM of three lodged sorghum plants (FPKM_lodged), and statistical significance (yes/no). (PDF 42 kb) [file 12870_2017_1218_MOESM3_ESM.pdf]

Calvin cycle

Gene name	Reaction EC	Enzymatic activity	FPKM_intact	FPKM_lodged	significant
Sb10g002960.1	2.2.1.1	transketolase	23.6	32.7	no
Sb09g020140.1	2.2.1.1	transketolase	183.8	137.7	no
Sb02g005410.1	2.2.1.1	transketolase	0.2	1.8	yes
Sb02g005380.1	2.2.1.1	transketolase	0.3	2.0	yes
Sb10g002960.1	2.2.1.1	transketolase	23.6	32.7	no
Sb09g020140.1	2.2.1.1	transketolase	183.8	137.7	no
Sb02g005410.1	2.2.1.1	transketolase	0.2	1.8	yes
Sb02g005380.1	2.2.1.1	transketolase	0.3	2.0	yes
Sb10g002220.1	2.2.1.1	transketolase	184.9	132.2	no
Sb10g002250.1	2.2.1.1	transketolase	1.0	0.5	no
Sb09g005230.1	2.2.1.1	transketolase	8.7	22.3	yes
Sb06g004280.1	2.2.1.1	transketolase	1.0	1.8	no
Sb10g002220.1	2.2.1.1	transketolase	184.9	132.2	no
Sb10g002250.1	2.2.1.1	transketolase	1.0	0.5	no
Sb09g005230.1	2.2.1.1	transketolase	8.7	22.3	yes
Sb06g004280.1	2.2.1.1	transketolase	1.0	1.8	no
Sb01g033450.1	2.7.1.19	phosphoribulokinase	17.0	23.8	no
Sb06g012270.1	2.7.1.19	phosphoribulokinase	3.0	5.5	no
Sb06g027270.1	2.7.1.19	phosphoribulokinase	3.1	1.7	no
Sb04g030950.1	2.7.1.19	phosphoribulokinase	205.9	130.2	yes
Sb03g037165.1	2.7.2.3	phosphoglycerate kinase	0.0	0.0	no
Sb01g020930.1	2.7.2.3	phosphoglycerate kinase	1.5	1.9	no
Sb04g004690.1	2.7.2.3	phosphoglycerate kinase	574.7	535.9	no
Sb09g024340.1	2.7.2.3	phosphoglycerate kinase	102.8	77.2	no
Sb10g026710.1	2.7.2.3	phosphoglycerate kinase	41.1	60.9	no
Sb10g026540.1	3.1.3.11	fructose-1,6-bisphosphatase	8.1	9.1	no
Sb09g021620.1	3.1.3.11	fructose-1,6-bisphosphatase	83.5	39.2	yes
Sb03g040900.1	3.1.3.11	fructose-1,6-bisphosphatase	147.7	61.6	yes
Sb01g039980.1	3.1.3.11	fructose-1,6-bisphosphatase	51.0	34.9	yes
Sb08g002160.1	4.1.1.39	ribulose-bisphosphate carboxylase	14.0	13.8	no
Sb01g044490.1	4.1.1.39	ribulose-bisphosphate carboxylase	32.0	23.8	no
Sb05g003480.1	4.1.1.39	ribulose-bisphosphate carboxylase	645.4	720.1	no
Sb04g001123.1	4.1.2.13	fructose-bisphosphate aldolase	21.3	18.3	no
Sb01g046740.1	4.1.2.13	fructose-bisphosphate aldolase	14.5	19.6	no
Sb01g021020.1	4.1.2.13	fructose-bisphosphate aldolase	58.5	58.3	no
Sb10g023850.1	4.1.2.13	fructose-bisphosphate aldolase	2.7	2.0	no
Sb08g004500.1	4.1.2.13	fructose-bisphosphate aldolase	424.4	300.1	no
Sb05g004590.1	4.1.2.13	fructose-bisphosphate aldolase	215.5	54.5	yes
Sb04g019020.1	4.1.2.13	fructose-bisphosphate aldolase	115.9	151.9	no
Sb03g008050.1	4.1.2.13	fructose-bisphosphate aldolase	75.7	106.2	no
Sb03g043140.1	4.1.2.13	fructose-bisphosphate aldolase	768.0	1244.6	no
Sb02g029250.1	5.1.3.1	ribulose-phosphate 3-epimerase	74.6	81.3	no
Sb01g045920.1	5.1.3.1	ribulose-phosphate 3-epimerase	195.0	186.6	no
Sb03g006130.1	5.3.1.1	ribulose-phosphate 3-epimerase	430.7	546.3	no
Sb03g039480.1	5.3.1.1	ribulose-phosphate 3-epimerase	17.4	8.4	yes
Sb02g031030.1	5.3.1.1	ribulose-phosphate 3-epimerase	148.0	125.8	no

Figure S2
