# Supplementary material for: Root lodging is a physical stress that changes gene expression from sucrose accumulation to degradation in sorghum
Source: BMC Plant Biol. 2018 Jan 3;18:2. doi: 10.1186/s12870-017-1218-9 (PMC5751775; doi:10.1186/s12870-017-1218-9)
Supplement: Supplementary file 4 — Comparison of expression levels of genes involved in C4 photosynthesis in intact and lodged stems. Designations are as in Additional file 3: Figure S2. (PDF 37 kb) [file 12870_2017_1218_MOESM4_ESM.pdf]

C4 photosynthesis

Gene name	Reaction EC	Enzymatic activity	FPKM_intact	FPKM_lodged	significant
Sb10g021330.1	4.1.1.31	phosphoenolpyruvate carboxylase	142.3	105.8	no
Sb07g014960.1	4.1.1.31	phosphoenolpyruvate carboxylase	4.3	14.6	yes
Sb04g008720.1	4.1.1.31	phosphoenolpyruvate carboxylase	142.7	125.0	no
Sb03g008410.1	4.1.1.31	phosphoenolpyruvate carboxylase	0.1	0.1	no
Sb03g035090.1	4.1.1.31	phosphoenolpyruvate carboxylase	22.5	23.2	no
Sb02g021090.1	4.1.1.31	phosphoenolpyruvate carboxylase	36.9	89.8	yes
Sb07g023910.1	1.1.1.82	malate dehydrogenase 1	33.0	21.4	yes
Sb07g023920.1	1.1.1.82	malate dehydrogenase 1	167.1	132.9	no
Sb09g019930.1	2.7.9.1	pyruvate phosphate dikinase	437.0	354.4	no
Sb01g031660.1	2.7.9.1	pyruvate phosphate dikinase	3.3	4.7	no

Figure S3
